# Prehospital management of chest injuries in severely injured patients—a systematic review and clinical practice guideline update

**DOI:** 10.1007/s00068-024-02457-3

**Published:** 2024-02-03

**Authors:** Christian Waydhas, Barbara Prediger, Oliver Kamp, Christian Kleber, André Nohl, Stefan Schulz-Drost, Christof Schreyer, Robert Schwab, Manuel Florian Struck, Jessica Breuing, Heiko Trentzsch

**Affiliations:** 1 Department of Trauma, Hand and Reconstructive Surgery, Essen University Hospital, Essen, Germany; 2 Department of Surgery, BG Bergmannsheil University Hospital, Bochum, Germany; 3 Institute for Research in Operative Medicine, Witten/Herdecke University, Cologne, Germany; 4 Department of Orthopaedics, Trauma Surgery and Plastic Surgery, Leipzig University Hospital, Leipzig, Germany; 5 Centre of Emergency Medicine, BG Duisburg Hospital, Duisburg, Germany; 6 Zentrum für Bewegungs- und Altersmedizin, Helios Kliniken Schwerin, Schwerin, Germany; 7 Department für Unfall- und Orthopädische Chirurgie, Universitätsklinikum Erlangen, Friedrich-Alexander-Universität Erlangen-Nürnberg, Erlangen, Germany; 8 Department of General, Visceral and Thoracic Surgery, Bundeswehr Central Hospital, Koblenz, Germany; 9 Department of Anaesthesiology and Intensive Care Medicine, Leipzig University Hospital, Leipzig, Germany; 10 Institute of Emergency Medicine and Medical Management, LMU Munich University Hospital, Munich, Germany

**Keywords:** Prehospital trauma management, Chest injury, Pneumothorax, Diagnostic test accuracy, Polytrauma guideline, Multiple injuries

## Abstract

**Purpose:**

Our aim was to review and update the existing evidence-based and consensus-based recommendations for the management of chest injuries in patients with multiple and/or severe injuries in the prehospital setting. This guideline topic is part of the 2022 update of the German Guideline on the Treatment of Patients with Multiple and/or Severe Injuries.

**Methods:**

MEDLINE and Embase were systematically searched to May 2021. Further literature reports were obtained from clinical experts. Randomised controlled trials, prospective cohort studies, cross-sectional studies, and comparative registry studies were included if they compared interventions for the detection and management of chest injuries in severely injured patients in the prehospital setting. We considered patient-relevant clinical outcomes such as mortality and diagnostic test accuracy. Risk of bias was assessed using NICE 2012 checklists. The evidence was synthesised narratively, and expert consensus was used to develop recommendations and determine their strength.

**Results:**

Two new studies were identified, both investigating the accuracy of in-flight ultrasound in the detection of pneumothorax. Two new recommendations were developed, one recommendation was modified. One of the two new recommendations and the modified recommendation address the use of ultrasound for detecting traumatic pneumothorax. One new good (clinical) practice point (GPP) recommends the use of an appropriate vented dressing in the management of open pneumothorax. Eleven recommendations were confirmed as unchanged because no new high-level evidence was found to support a change.

**Conclusion:**

Some evidence suggests that ultrasound should be considered to identify pneumothorax in the prehospital setting. Otherwise, the recommendations from 2016 remained unchanged.

**Supplementary Information:**

The online version contains supplementary material available at 10.1007/s00068-024-02457-3.

## Introduction

In Germany, the chest is the body region that is most affected in polytrauma patients. A serious injury, i.e. an injury with an Abbreviated Injury Scale score of at least 3, is present in 46.1% of patients [1]. Several thoracic injuries can lead to acute life-threatening conditions that, based on the ABCDE priorities of trauma management, affect a patient’s breathing (B) or circulation (C). These conditions and their immediate consequences can develop dynamically and must therefore be expected to occur even in the prehospital setting. Since qualified emergency medical services (EMS) personnel usually arrive at the scene of an incident at an early stage, it is possible that life-threatening conditions and their causes can be detected, and appropriate life-saving interventions can be performed in this setting. The objective of this systematic review is to identify the current evidence on diagnostic and treatment approaches especially in the prehospital setting and to assess its usefulness as a basis for evidence-based clinical recommendations for emergency physicians and EMS personnel.

## Methods

This guideline topic is part of the 2022 update of the German Guideline on the Treatment of Patients with Multiple and/or Severe Injuries [2]. The guideline update is reported according to the RIGHT tool [3], the systematic review part according to the Preferred Reporting Items for Systematic Reviews and Meta-Analyses (PRISMA) 2020 reporting guideline [4]. The development and updating of recommendations followed the standard methodology set out in the guideline development handbook issued by the German Association of the Scientific Medical Societies (AWMF) [5]. All methods were defined a priori, following the methods report of the previous guideline version from July 2016 [6, 7] with minor modifications, as detailed below. The “Discussion” section of this publication is a direct translation of the original guideline text [2].

### PICO questions and eligibility criteria

Population, intervention, comparison, and outcome (PICO) questions were retained from the previous guideline version. In addition, the participating professional societies involved in guideline development were asked to submit new PICO questions. The overarching PICO question for this topic area was:

In adult patients (≥14 years) with known or suspected polytrauma and/or severe injuries, does a specific prehospital approach to the management of chest injuries improve patient-relevant outcomes compared to any other intervention?

The full set of predefined PICO questions is listed in Table S1 (Online Resource). The study selection criteria in the PICO format are shown in Table 1.

**Table 1:** Predefined selection criteria

Population:	Adult patients (≥ 14 years) with polytrauma and/or severe injuries^a^
Intervention/comparison:	Prehospital management of chest injuries
Outcomes:	Any patient-relevant outcome, such as prehospital and hospital mortality, immediate complications, long-term adverse effects, and diagnostic test accuracy
Study type:	• Comparative, prospective studies (randomised controlled trials, cohort studies) • Comparativeregistry^b^ data (incl. case–control studies) • Cross-sectional studies (only diagnostic studies) • Systematic reviews based on the above primary study types
Language:	English or German
Other inclusion criteria:	• Full Text Of Study Published And Accessible • Study matches predefined PICO question
Exclusion criteria:	• Multiple publications of the same study without additional information

^a^Defined by an Injury Severity Score (ISS) > 15, Glasgow Coma Scale (GCS) < 9, or comparable values on other scales, or, in the prehospital setting, clinical suspicion of polytrauma/severe injury with a need for life-saving interventions

^b^Using the Agency for Healthcare Research and Quality (AHRQ) definition of registries [8]

### Literature search

An information specialist systematically searched for literature in MEDLINE (Ovid) and Embase (Elsevier). The search strategy described in the 2011 Guideline was used with modifications. It contained index (MeSH/Emtree) and free-text terms for the population and intervention. The searches were completed on 19 May 2021. The start date for update searches was 1 June 2014. Table S2 (Online Resource) provides details for all searches. Searches were conducted for both prehospital and inhospital care. Clinical experts were asked to submit additional relevant references.

### Study selection

Study selection was performed independently by two reviewers in a two-step process using the predefined eligibility criteria: (1) title/abstract screening of all references retrieved from database searches using Rayyan software [9] and (2) full-text screening of all articles deemed potentially relevant by at least one reviewer at the title/abstract level in Endnote (Endnote, Version: 20 [Software]. Clarivate, Boston, Massachusetts, USA; https://endnote.com/). Disagreements were resolved through consensus or by consulting a third reviewer. The reasons for full-text exclusion were recorded (Table S3, Online Resource).

### Assessment of risk of bias and level of evidence

Two reviewers sequentially assessed the risk of bias of included studies at study level using the relevant checklists from the NICE guidelines manual 2012 [10] and assigned each study an initial level of evidence (LoE) using the Oxford Centre for Evidence-based Medicine Levels of Evidence (2009) [11]. Any disagreements were resolved through consensus or by consulting a third reviewer.

### Data extraction and data items

Data were extracted into a standardised data table by one reviewer and checked by another. A predefined dataset was collected for each study, consisting of study characteristics (study type, aims, setting), patient selection criteria and baseline characteristics (age, gender, injury scores, other relevant variables), intervention and control group treatments (including important co-interventions, index and reference tests for diagnostic studies), patient flow (number of patients included and analysed), matching/adjusting variables, and data on outcomes for any time point reported.

### Outcome measures

Outcomes were extracted as reported in the study publications. For prospective cohort studies and registry data, preference was given to data obtained after propensity-score matching or statistical adjustment for risk-modulating variables over unadjusted data.

### Synthesis of studies

Studies were grouped by interventions. An interdisciplinary expert group used their clinical experience to synthesise studies narratively by balancing beneficial and adverse effects extracted from the available evidence. Priority was given to diagnostic test accuracy, reducing prehospital and inhospital mortality, immediate complications, and long-term adverse effects. Clinical heterogeneity was explored by comparing inclusion criteria and patient characteristics at baseline as well as clinical differences in the interventions and co-interventions.

### Development and updating of recommendations

For each PICO question, the following updating options were available: (1) the recommendation of the preceding version remains valid and requires no changes (“confirmed”); (2) the recommendation requires modification (“modified”); (3) the recommendation is no longer valid or required and is deleted; (4) a new recommendation needs to be developed (“new”). An interdisciplinary expert group of clinicians with expertise in trauma and acute care (comprising prehospital emergency physicians, anaesthesiologists, trauma surgeons, thoracic surgeons) reviewed the body of evidence, drafted recommendations based on the homogeneity of clinical characteristics and outcomes, the balance between benefits and harms, as well as their clinical expertise, and proposed grades of recommendation (Table 2). In the absence of eligible evidence, recommendations were made based on clinical experience, data from studies with a lower level of evidence, and expert consensus in cases where the Guideline Group felt a statement was required due to the importance of the topic. These were not graded, and instead labelled as good (clinical) practice points (GPP). For GPPs, the strength of a recommendation is presented in the wording shown in Table 2.

**Table 2:** Grading of recommendations

Symbol	Grade of recommendation	Description	Wording (examples)
⇑⇑	A	Strong recommendation	“use …”, “do not use …”
⇑	B	Recommendation	“should use …”, “should not use …”
⇔	0	Open recommendation	“consider using …”, “… can be considered”

### Consensus process

The Guideline Group finalised the recommendations during web-based, structured consensus conferences on 13 September 2021 and 15 March 2022 via Zoom (Zoom, Version: 5.x [Software], Zoom Video Communications, Inc., San José, California, USA; https://zoom.us). A neutral moderator facilitated the consensus conference. Voting members of the Guideline Group were delegates of all participating professional organisations, including clinicians, emergency medical services personnel, and nurses, while guideline methodologists attended in a supporting role. Members with a moderate, thematically relevant conflict of interest abstained from voting on recommendations; members with a high, relevant conflict of interest were not permitted to vote or participate in the discussion. Attempts to recruit patient representatives were unsuccessful. A member of the expert group presented recommendations. Following discussion, the Guideline Group refined the wording of the recommendations and modified the grade of recommendation as needed. Agreement with both the wording and the grade of recommendation was assessed by anonymous online voting using the survey function of Zoom. Abstentions were subtracted from the denominator of the agreement rate. Consensus strength was classified as shown in Table 3.

**Table 3:** Classification of consensus strength

Description	Agreement rate
Strong consensus	> 95% of participants
Consensus	> 75 to 95% of participants
Majority approval	> 50 to 75% of participants
No approval	< 50% of participants

Recommendations were accepted if they reached consensus or strong consensus. For consensus recommendations with ≤ 95% agreement, diverging views by members of the Guideline Group were detailed in the background texts. Recommendations with majority approval were returned to the expert group for revision and further discussion at a subsequent consensus conference. Recommendations without approval were considered rejected.

### External review

During a 4-week consultation phase, the recommendations and background texts were submitted to all participating professional organisations for review. Comments were collected using a structured review form. The results were then assessed, discussed, and incorporated into the text by the guideline coordinator with the relevant author group.

The guideline was adopted by the executive board of the German Trauma Society on 17 January 2023.

### Quality assurance

The guideline recommendations were reviewed for consistency between guideline topic areas by the steering group. Where necessary, changes were made in collaboration with the clinical leads for all topic areas concerned. The final guideline document was checked for errors by the guideline chair and methodologist.

## Results

**Fig. 1:**
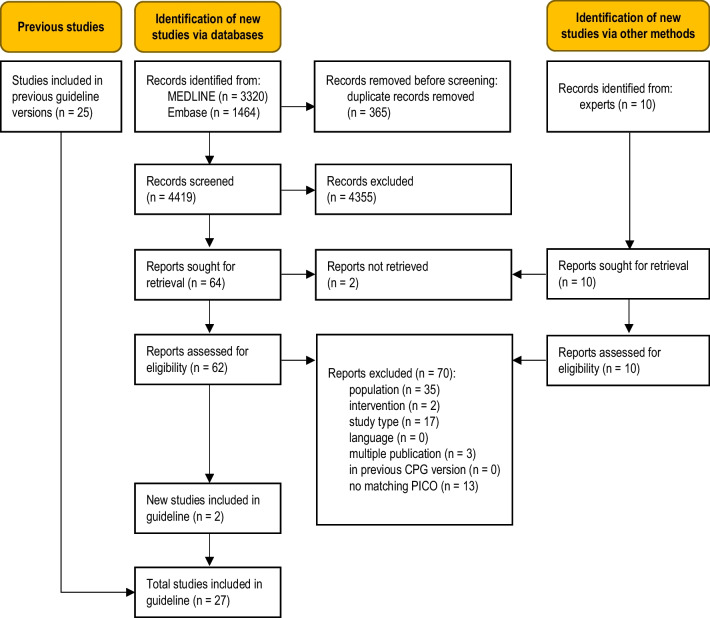
Modified PRISMA 2020 flow diagram showing the systematic literature search and selection of studies

The database searches identified 4419 unique records (Fig. 1). Ten additional records were obtained from clinical experts. Two studies were eligible for this update [12, 13]. A total of 70 full-text articles were excluded (Table S3, Online Resource).

### Characteristics of studies included in this update

Study characteristics, main outcomes, levels of evidence, and risk-of-bias assessments are presented in Table 4. Full details are provided in Table S4 (Online Resource). The evidence included two diagnostic accuracy studies. Both studies were performed in the USA. Eligible patient populations were adults with severe injuries and suspected injuries to the chest. The detection of pneumothorax using in-flight ultrasonography was studied in both publications.

**Table 4:** Characteristics of studies included in the update

Study, reference	Design	Setting	Population	Age, ISS^*^	Interventions (*N* patients)	Main outcomes (selection)*	LoE, risk of bias (RoB)^§^, comments
*Suspected diagnosis of pneumothorax and/or haemothorax*							
Press 2014 [12]	Diagnostic cross-sectional study	USA, 7-month period	Trauma patients	*Age [y], mean* ± *SD* 41 ± 17 *Mean ISS* ± *SD* 16 ± 11	*N* = 833 Patients with at least one ultrasound examination performed by HEMS providers (*N* = 293) Number of lung ultrasound examinations performed by HEMS providers (*N* = 511) Index test: in-flight ultrasound Reference standard: diagnostic procedures and management in the ED including CT, chest radiography and clinical examination	Diagnostic test performance Pneumothorax *Sensitivity, % (95% CI), n/N* 18.7 (8.9–33.9), 8/43 *Specificity, % (95% CI), n/N* 99.5 (98.2–99.9), 444/446	LoE 2b low RoB
Quick 2016 [13]	Diagnostic cross-sectional study	USA, 15-month period	Trauma patients	*Age [y], mean (range)* 44.4 (18–94) *Mean ISS (range)* 17.68 (1–75)	Index test: in-flight ultrasound (*N* = 149) Reference standard: CT scan (*N* = 116)	Diagnostic test performance Pneumothorax *Diagnostic accuracy, % (95% CI)* 91 (0.85–0.95) *Sensitivity, % (95% CI)* 68 (0.46–0.85) *Specificity, % (95% CI)* 96 (CI 0.90–0.98)	LoE 2b unclear RoB

*CT *computed tomography, *d *days, *ED *emergency department,* h *hours, *HEMS *helicopter emergency medical services, *HR *hazard ratio, *ISS *injury severity score, *SD *standard deviation, *y y*ears, *CI* confidence interval, *IG *intervention group, *CG *control group

*Data for IG versus CG unless otherwise specified

^§^Risk of bias: *low RoB*, RoB low for all domains; *unclear RoB*, RoB unclear for at least one domain, no high RoB in any domain; for studies with high RoB, all domains with high RoB are named, with RoB low or unclear for all other domains (for full details Table S4, Online Resource 1)

### Risk-of-bias assessment for included studies and levels of evidence

One study was judged to be of low risk of bias in all domains. The risk of bias regarding flow and timing was unclear in one study.

### Recommendations

One recommendation was modified and one new recommendation as well as one new GPP were developed based on the updated evidence and expert consensus. Eleven recommendations were confirmed as unchanged because no new high-level evidence was found to support a change (Table 5). All achieved strong consensus. One recommendation from the 2016 Guideline was not retained in the 2022 update (Table S5, Online Resource).

**Table 5:** List of recommendations with grade of recommendation and strength of consensus

No	GoR	Evidence, consensus	Recommendation	Status 2022
*Diagnosis*				
1	A ⇑⇑	100%	Perform a clinical examination of the chest and respiratory function	Confirmed
2	B ⇑	100%	The examination should include at least respiratory rate measurement and lung auscultation. Repeated examinations should follow	Confirmed
3	0 ⇔	100%	Inspection, palpation, and percussion of the chest as well as pulse oximetry and monitoring of ventilatory pressure and capnography in ventilated patients can be useful	Confirmed
4	0 ⇔	[12, 13] 100%	Chest ultrasonography may be performed to detect or rule out pneumothorax or pericardial effusion	New
5	A ⇑⇑	100%	Make a diagnosis of pneumothorax and/or haemothorax in patients with decreased or absent unilateral breath sounds (after confirmation of correct tube position) or in the presence of ultrasonographic signs	Modified
6	B ⇑	100%	Possible progression of a small pneumothorax that cannot initially be detected in the prehospital setting should be borne in mind	Confirmed
7	B ⇑	100%	A diagnosis of tension pneumothorax should be suspected in patients with unilaterally absent breath sounds on auscultation (after confirmation of correct tube position) and in the additional presence of typical signs and symptoms, especially those of severe respiratory or circulatory compromise	Confirmed
*Treatment*				
8	A ⇑⇑	100%	Decompress a clinically suspected tension pneumothorax immediately	Confirmed
9	B ⇑	100%	Pneumothorax diagnosed by auscultation should be decompressed in patients on positive pressure ventilation	Confirmed
10	B ⇑	100%	Pneumothorax diagnosed on auscultation should be managed by observation and close monitoring in patients who are not mechanically ventilated	Confirmed
11	B ⇑	100%	Tension pneumothorax should be managed by needle decompression (one attempt only) or immediate mini-thoracotomy. Needle decompression should be followed by a surgical incision into the pleural space with or without chest tube insertion	Confirmed
12	B ⇑	100%	Pneumothorax should be managed by chest tube placement, if indicated	Confirmed
13	B ⇑	100%	Surgical access to the pleural space should be achieved by mini-thoracotomy. Chest tubes should be placed without a trocar	Confirmed
14	GPP	100%	Open pneumothorax should be managed using an appropriate vented dressing	New

*GoR*, grade of recommendation

## Discussion and Rationale for Recommendations

### Diagnosis

Physical examination supported using EMS technical equipment is essential for the formulation of a (working) diagnosis [7, 14–16]. Decisions about any necessary treatment cannot be made without a diagnosis. There is a lack of comprehensive evidence regarding the relative role of individual diagnostic measures. Nevertheless, chest auscultation, respiratory rate measurement, and the assessment of spontaneous pain and pain/tenderness on palpation have been shown to have sufficient diagnostic accuracy and to provide information that is relevant for treatment in the acute setting [7, 14, 17–20]. Other types of examination (palpation, percussion) can be used in individual cases, but their accuracy and relevance are not well studied [7]. Continuous monitoring using pulse oximetry and capnography (in ventilated patients) together with repeated physical examinations may help detect dynamic deterioration. Progression of pneumothorax (or another chest injury) should be considered a possible cause of deterioration.

Small mobile ultrasound devices have become increasingly available for use in the prehospital care setting (and their quality has progressively improved over the years). A Cochrane review has shown that the sensitivity of ultrasound in trauma patients (91%) was almost twice as high as that of standard chest X-ray (47%) in hospital emergency departments [21]. Compared with computed tomography, the sensitivity of ultrasound was still 81% [22]. The specificity of ultrasound was found to be 98–99%.

Different results regarding the use of ultrasonography in the prehospital setting were reported in two prospective observational studies. In the earlier study, which was published in 2014, ultrasonography for pneumothorax had a sensitivity of only 18.7% and a specificity of 99.5% [23]. In the study by Quick et al. [13], ultrasonography correctly identified 68% of pneumothoraces and had a specificity of 96%. Positive predictive values of 80% and 94.2% and negative predictive values of 92.7% and 97.7% were obtained. Diagnostic accuracy in the prehospital setting was thus slightly lower than that reported for the resuscitation room setting. It was, however, still higher than the diagnostic accuracy of a purely clinical examination. This applies to patients with decreased levels of consciousness. A possible explanation for this slightly poorer result may be that examinations in the prehospital setting take place in less favourable conditions (lighting, time pressure, weather, and other environmental conditions, etc.). Moreover, selection bias was present in both studies and different reference standards (chest computed tomography, standard chest X-ray, and clinical examination) were used. In both studies, ultrasound examinations were performed by EMS personnel (with special training in ultrasound but mostly without previous experience in ultrasound imaging). By contrast, ultrasound is predominantly used by emergency physicians in Germany. Whether emergency physicians have better ultrasound skills than EMS personnel is unknown. Although ultrasound devices have been available to air medical services for many years, there are no systematic reports or clinical studies with large sample sizes. Inhospital results cannot be simply transferred to the prehospital setting. In the inhospital setting, ultrasound examinations are often performed by radiologists. The expertise of emergency physicians and EMS personnel is unknown.

Likewise, it is completely unclear how often ultrasonographic evidence of the presence or absence of pneumothorax results in, or would result in, therapeutic consequences and better outcomes. A benefit-risk analysis cannot be carried out based on current knowledge.

As long as there are no data from Germany or similar emergency medical service systems which provide reliable evidence for a broad competence in prehospital ultrasound among emergency physicians and for therapeutic consequences of ultrasonographic findings that lead to a positive benefit-risk assessment, there is no basis for a higher grade of recommendation.

### Diagnosis of pneumothorax

In patients with unilaterally decreased breath sounds in association with shortness of breath and/or chest pain, the probability of pneumothorax is 90–99% [14]. If none of these three signs and symptoms is present, the probability of pneumothorax is less than 1%. This also means that a major pneumothorax can be largely ruled out in the absence of these auscultation findings, especially in patients with normal breathing and no chest pain. In patients with detected pneumothorax but equal bilateral breath sounds, the mean volume of pneumothorax was reported to be 378 mL (maximum volume, 800 mL) [24]. When auscultation findings are interpreted, correct positioning of the endotracheal tube (if present) must be ensured as far as possible. The specificity and positive predictive value of soft tissue emphysema or flail chest are unknown [14]. The presence of bilateral pneumothorax must be considered in patients with severe bilateral chest trauma and may be associated with atypical examination results. Data that help differentiate between a pneumothorax and a haemothorax, or a combination of both, are not available. Percussion can be helpful but is of limited relevance in the prehospital setting since the differentiation between pneumothorax and haemothorax does not have a verifiable effect on treatment needs.

If left untreated, pneumothorax can progress to tension pneumothorax. Even occult pneumothoraces can progress to pneumothoraces that require chest tube drainage in 6–9.5% [25–30] and in as much as 14% of ventilated patients [29]. It can be assumed that the rate of progression is higher for large pneumothoraces, but this is not definitely known. The main risk is that an originally asymptomatic pneumothorax progresses to a tension pneumothorax.

### Diagnosis of tension pneumothorax

The signs and symptoms and the order of occurrence of signs and symptoms differ between patients who are breathing unassisted and patients receiving assisted ventilation [31]. Respiratory distress and tachycardia are the usual predominant presenting features of tension pneumothorax in awake, spontaneously ventilating patients [15]. Chest pain, tachypnoea, and decreased breath sounds were reported to be present in more than 45% of spontaneously breathing patients. Dyspnoea/respiratory distress, hypoxia and requirement for supplemental oxygen, tachycardia, and percussion hyperresonance were noted in 30 to 45%. Contralateral tracheal deviation (15–30%) or hypotension (developing in a relatively delayed fashion), jugular venous distention, subcutaneous emphysema, and cardiac arrest (each in less than 15%) were less common [31]. Experimental studies have shown that respiratory distress and paralysis of the respiratory centre as a result of hypoxia precede cardiac arrest in awake patients and that hypotension progressing to cardiac arrest is a late sign of tension pneumothorax [32, 33].

By contrast, the haemodynamic consequences of tension pneumothorax (e.g. decrease in blood pressure, shock) were reported to occur much earlier in ventilated patients and usually at the same time as respiratory signs and symptoms [15, 31]. Decreased breath sounds, hypotension (often with sudden onset), and hypoxia were noted with equal frequency (> 45%). Tachycardia, subcutaneous emphysema, and cardiac arrest were common as well (30–45%) [31]. In ventilated patients, considerably elevated or increasing airway pressure is another symptom that is seen in about 20% of patients with haemothorax/pneumothorax [16, 17].

According to expert opinion, the presence of tension pneumothorax is highly probable in patients with a combination of (unilaterally) absent breath sounds (after confirmation of correct tube position) and life-threatening respiratory or cardiovascular dysfunction so that the diagnosis should be established based on these findings and the necessary therapeutic measures should be initiated. Further additional diagnostic procedures should not be undertaken since they would present an avoidable delay. The consequences of a false positive diagnosis of tension pneumothorax appear to be less severe than those of not performing any necessary decompression.

### Treatment of pneumothorax and tension pneumothorax (indications for treatment)

Tension pneumothorax is an acute life-threatening condition and, if left untreated, usually leads to death. Death can occur within a few minutes of the onset of signs and symptoms of impaired pulmonary and cardiovascular function. There is no alternative to decompression. Expert opinion is that emergency decompression must be immediately performed especially in patients with haemodynamic or respiratory compromise and that patient transport, even to a nearby hospital, would cause an unacceptable delay. A study involving 3500 autopsy records identified 39 cases of tension pneumothorax (incidence, 1.1%), half of which had not been diagnosed while the patients were still alive. Among soldiers serving in the Vietnam War, tension pneumothorax was detected in 33% of patients with fatal chest injuries [34]. An analysis of 20 patients who had been identified as unexpected survivors based on the Trauma and Injury Severity Score (TRISS) showed that seven of these 20 patients had undergone prehospital decompression for tension pneumothorax [35]. Four of 18 trauma patients who had undergone resuscitation in the prehospital setting had a return of cardiac output following decompression [36]. An analysis of patients with traumatic cardiac arrest identified decompression of tension pneumothorax as the most important factor contributing to an improvement in prognosis [37]. In recent analyses, too, untreated tension pneumothorax has been identified as one of the most common causes of potentially preventable deaths [38].

A large pneumothorax, which must be assumed in the presence of typical auscultation findings, is an indication for pleural space evacuation. Whether this must be done in the prehospital or in the hospital setting is difficult to decide in individual cases since the risk of progression from simple pneumothorax to tension pneumothorax and the period over which progression can take place vary and are difficult to estimate. Ventilated patients have a considerably higher risk of pneumothorax progression [39]. According to expert opinion, it is therefore plausible that a pneumothorax detected by auscultation in ventilated patients is associated with a considerably elevated risk of developing into a tension pneumothorax and thus is an indication for prehospital decompression. Conversely, pneumothoraces develop into tension pneumothoraces in less than 10% of spontaneously breathing patients [39]. Based on a benefit-risk assessment, prehospital decompression therefore appears to be unnecessary in patients with no or mild signs and symptoms and no signs and symptoms of progression. These patients must be managed by observation, close monitoring, and clinical reevaluation. If patients cannot be easily monitored and clinically reviewed, for example during helicopter transport, there is a certain non-quantifiable risk that a tension pneumothorax develops and that this condition is not detected in time or that there is not enough space to provide appropriate treatment. In such situations, and depending on individual circumstances, decompression of pneumothorax may also be performed in non-intubated patients with relevant clinical signs and symptoms prior to transport.

If equal bilateral breath sounds are found, a clinically relevant pneumothorax will unlikely be present. This means there is no indication for prehospital decompression or pleural space evacuation even if other signs of chest trauma (but no specific signs of pneumothorax) are present. In a systematic review [14], the incidence of pneumothorax was reported to be relatively low despite the presence of chest trauma (10 to 50%). This means that if there is no clear evidence of pneumothorax and an intervention is performed based on a diagnosis of chest trauma alone, at least every second patient or even up to nine out of 10 patients will undergo an unnecessary invasive procedure. Since relevant studies also included occult pneumothoraces that were detected only via computed tomography, the percentage of cases requiring chest tube drainage may be even lower. Even when pneumothorax was suspected based on specific clinical signs, the rate of unnecessary needle decompressions and chest tube drainage ranged between 9 and 65% [17, 40, 41]. Under these conditions, there is no indication for decompression in non-ventilated patients.

In general, chest drainage for haemothorax is not indicated in the prehospital setting. Although a large haemothorax (approximately > 300 mL) is usually an indication for the drainage of blood from the pleural cavity, the space-occupying effect of an accumulation of blood does not pose a direct danger unless the rare situation of a tension haemothorax is present. Only then is emergency chest drainage indicated. Tension haemothorax, however, commonly presents with similar signs and symptoms as tension pneumothorax (absent breath sounds on one side and severe respiratory and/or circulatory compromise).

### Management of pneumothorax and tension pneumothorax

Tension pneumothorax can be treated with needle decompression, simple thoracostomy (without chest tube insertion), or tube thoracostomy. There are no comparative studies providing evidence of the superiority of one of these three modes of treatment.

From a pathophysiological perspective, sustained decompression requires that the amount of air that enters the pleural space during a spontaneous or mechanical breath be removed through the drainage device (regardless of the method used). Volumetric flow is proportional to the fourth power of the internal diameter of the needle. Needle decompression (or even single chest tube insertion, e.g. for the management of tracheobronchial injuries) can thus be ineffective if the lumen is too small for adequate drainage.

As a result of the low level of evidence for the various methods and benefit-risk profiles that can be used as a basis for a direct comparison of methods, treatment decisions should address practical aspects and potential risks and should therefore also consider the skills of prehospital care providers and the availability of drainage devices.

### Thoracostomy

Chest tube insertion not only is an appropriate and highly effective (> 85%) procedure for the decompression of tension pneumothorax but is also associated with complications. It must be used when other measures fail or are inadequate. Usually, it is a definitive procedure and has the highest success rate. Prehospital pleural drainage was successfully used as a definitive procedure in 79–95% of cases [7, 14]. At the same time, pleural drainage was reported to have a mean failure rate of 11.2% because of incorrect positioning or insufficient effectiveness of the tube [7, 14] so that additional chest tube insertion was required. Chest tubes that are inserted in the prehospital setting are associated with significantly higher complication rates than those inserted in the hospital setting. Complications included subcutaneous tube placement (2.53% versus 0.39%), intraparenchymal tube placement (1.37% versus 0.63%), and intra-abdominal tube misplacement (0.87% versus 0.73%) [7, 14].

Simple thoracostomy (without chest tube insertion) is an appropriate, effective, and relatively rapid procedure for tension pneumothorax decompression. This technique, however, can only be used for patients undergoing positive pressure ventilation since pressure in the intrapleural space never becomes negative in these patients. Spontaneous ventilation generates negative intrapleural pressures that may be sufficient to suck air through the thoracostomy into the pleural cavity. In a case series involving 45 patients, simple thoracostomy was used in the prehospital setting and was found to be an effective treatment that did not cause relevant complications [42]. In a prospective observational study, 55 patients with 59 suspected pneumothoraces were treated with simple thoracostomy by a helicopter emergency medical service over a period of 2 years. Mean oxygen saturation increased after the procedure from 86.4 to 98.5%. A pneumothorax or a haemopneumothorax was found in 91.5% of the patients. The authors did not observe any cases of recurrent tension pneumothorax or major complications such as major bleeding, lung laceration, or pleural empyema [43]. A chest tube is then inserted through the thoracostomy in the hospital setting.

Controlled studies addressing the different techniques for chest tube insertion are not available. Most experts recommend the following standardised technique. Chest tubes must be placed using a sterile technique. Local and systemic analgesia must be used in patients who are not unconscious. A trocar should never be inserted blindly to guide the tube through the chest. Studies investigating the trocar technique reported higher complication rates than studies addressing the surgical technique (11.0% versus 1.6%) [7, 14]. In a prospective cohort study (of critically ill patients), the use of a trocar was also associated with a significantly higher rate of chest tube malposition [44].

Small catheters (≤ 14 Fr) are usually inserted using the Seldinger technique, and large-bore chest tubes using mini-thoracotomy. In addition, modified Seldinger techniques using consecutive dilation are also available for large-bore chest tubes [45, 46].

It is generally recommended that the tip of the chest tube be directed in a specific location: posterior and inferior chest tube placement for haemothorax, anterior and superior placement for pneumothorax. This recommendation has been challenged by a recent study that did not detect any effects of chest tube position on the rate of success (drainage of air and blood) [47].

Typical locations for chest tube placement in trauma patients are the second or third intercostal space (ICS) in the mid-clavicular line (MCL) and the fourth or fifth ICS in the mid-axillary line (MAL). Reliable data on differences in the success rates and complication rates for tube placement in the MCL (in the second or third ICS) versus tube placement in the MAL (in the region of the fifth ICS) are not available [7, 14]. For this reason, there is no site that can be recommended as the preferred location for tube placement.

Several recommendations regarding tube size can be provided. In a small randomised controlled study, tube-site pain was assessed immediately after tube insertion and on the two following days in patients with uncomplicated traumatic pneumothorax and no relevant haemothorax (patients were awake, able to consent, and haemodynamically stable). Patients who were treated with 14-Fr catheters had significantly lower tube-site pain scores than patients who were treated with 28-Fr chest tubes. Success and complication rates were similar in the two groups [48]. In a systematic review that was published in 2018 [49], no other randomised studies addressing traumatic pneumothorax were identified.

In a small, randomised study on patients with uncomplicated traumatic haemothorax (patients were awake, able to consent, and haemodynamically stable), failure rates were 10% for small-calibre (14-Fr) catheters and 17% for large-bore (28–32 Fr) chest tubes. There was no difference between the two groups in drainage volumes (600 mL versus 400 mL). Patients with 14-Fr catheters, however, reported a significantly better insertion experience [50]. These results confirmed trends that were reported in two retrospective studies [51, 52].

By contrast, all severely injured patients with haemothorax or haemopneumothorax (patients are haemodynamically unstable and unable to provide consent due to trauma) are treated with large-bore chest tubes [53–56]. These patients have an increased risk of a large fistula volume or a large amount of blood. Large-bore chest tubes are used to prevent chest tube blockage by blood clots. A prospective observational study involving a total of 353 chest tubes found no difference between smaller chest tubes (28–32 Fr) and larger chest tubes (> 36 Fr) in terms of retained haemothoraces and the need for additional tube insertion [57].

Patients who present with uncomplicated pneumothorax or haemothorax and who are haemodynamically stable and awake can be treated with 14-Fr drainage catheters. Patients who present with complicated pneumothorax or haemothorax and who are haemodynamically unstable or unable to provide consent due to trauma should be treated with larger chest tubes (24–32-Fr).

### Needle decompression

Needle decompression is an appropriate and simple drainage procedure that is often effective (approximately 32–53%) but not free of complications [7, 14]. If the procedure fails or is insufficient, surgical decompression and/or chest tube insertion must be performed immediately. A failure rate of 58% was reported in a swine model. Causes were mechanical failure (due to kinking, obstruction, or dislodgment) within 5 min of placement or inadequate relief of tension [58]. In prehospital studies, needle decompression resulted in the release of air in 32–47% and clinical improvement in 12–60% of the patients who underwent needle decompression [17, 59, 60]. In 40% of cases, inadequate relief of tension required chest tube insertion after needle decompression. In other prehospital studies [59, 61], 53–67% of all patients were treated by needle decompression and subsequent chest tube insertion in the prehospital setting.

In two studies, total time on scene was significantly shorter (approximately 5 min) for patients treated with needle decompression (20.3 min) than for patients who underwent chest tube placement (25.7 min) [17, 59]. The time interval between the decision to perform decompression and successful decompression is more important than total time on scene. Needle decompression is the fastest method even for well-established teams and experienced surgeons. This applies to cases where prehospital providers are faced with nonoptimal conditions and have no relevant experience in chest tube insertion. For this reason, needle decompression is recommended as the first and fastest treatment for life-threatening tension pneumothorax. Needle decompression also appears to be an appropriate initial procedure in specific situations, for example when a patient is trapped or must be managed in adverse conditions (e.g. in underground tunnels).

If the first attempt at needle decompression is unsuccessful, a second attempt should not be made. Instead, thoracostomy should be performed immediately.

The Guideline Group is of the opinion that definitive treatment with mini-thoracotomy and chest tube insertion should be performed as soon as possible after successful needle decompression. Reasons for this are possible dislodgment, kinking, or obstruction of the needle during treatment, (re)positioning or transport as well as insufficient decompression in patients with large fistula volumes on positive pressure ventilation. This opinion is supported by a study in which 85% of patients required tube thoracostomy after needle decompression because of persistent symptoms or persistent pneumothorax on imaging [62].

Data on the size or type of cannula to be used are not available. A cannula with a diameter as large as possible (14 gauge or 12 gauge) is usually recommended to allow as much air as possible to be released.

Typical locations are the second or third intercostal space (ICS) in the mid-clavicular line (MCL), the fourth or fifth ICS in the mid-axillary line (MAL), and the fourth or fifth ICS in the anterior axillary line (AAL). In a meta-analysis, the mean distance from the skin surface to the pleural space was reported to be 34 mm (CI, 28–41 mm) at the AAL and was thus the shortest distance when compared to other sites [63]. The mean distance was 40 mm (CI, 29–51 mm) at the MAL and 43 mm (CI, 39–47 mm) at the MCL. As a result of the high level of heterogeneity among the studies included, however, these differences were not significant. In some studies, the distance at the MCL was reported to be even shorter than that at the MAL [7, 14]. The distance from the skin surface to the pleural space is significantly longer in women than in men [7, 14, 63]. In addition, there is a significant direct correlation between this distance and body mass index [64–66].

The use of a 3.2-cm cannula was associated with a failure rate of 65% [67]. Failure rates were considerably lower when a 5-cm cannula was used at the AAL (13%), MAL (31%), and MCL (38%) [63]. A failure rate of 89% was observed when a 5-cm cannula was used in obese patients (BMI > 30) [66].

A meta-analysis including 18 studies found that a catheter of at least 6.44 cm in length would be theoretically required to ensure that 95% of the patients would have penetration of the pleural space at the site of needle decompression [68]. In a study based on magnetic resonance imaging data from 2574 healthy volunteers who were representative of the population as a whole, Hecker et al. [69] found that decompression at an anterior location (MCL) might theoretically be successful in about 85% of cases when a 6.5-cm needle was used and in about 90% when a 7-cm needle was used. An evaluation of possible injuries to organs was limited to the internal mammary artery. The risks to this artery were assessed as minimal. There is, however, no clinical evidence of an improved success rate or a possible increase in procedure-related complications.

Success rates must be weighed against possible complications. A model-based study showed that, when an anterior location was used, there was a strong trend to performing needle decompression medial to the midclavicular line, which risked injuries to the heart, internal thoracic vessels, or other mediastinal vessels [70].

The longer the needle, the higher the risk of injuries to deep structures [7, 14, 63]. In a study based on CT images, the use of an 8-cm cannula could have led to injuries to vital structures in 32% of cases [64]. Clinical studies assessing the actual risks and benefits of longer versus normal cannulas are not available. The use of a 4.5-cm or 5-cm needle appears to have a relatively low risk of injuring vital structures but fails in at least one-third of cases. A longer needle (8 cm) is likely to achieve successful decompression in a larger number of cases but has a higher risk of injuries to vital structures (especially in association with left-sided procedures and lateral approaches).

Firm recommendations as to the most appropriate location of needle decompression cannot be made. Decisions as to the use of the MCL or AAL should be based on a patient’s individual anatomical features. The needle should have a minimum length of 5 cm. The optimum length of the cannula cannot be definitively determined. The use of a shorter cannula is associated with greater safety than the use of a longer cannula, which, however, has a higher success rate. Especially cannulas with a length of 8 cm or more must be expected to cause injuries to vital organs in up to 32% of cases [39].

### Open pneumothorax

Experimental studies have shown that open pneumothorax or haemopneumothorax can be successfully managed with appropriate dressings. These dressings usually allow air and blood to escape from the chest and can thus prevent the development of a tension pneumothorax [71–74]. There is concern that dressings can fail due to poor adherence to wet skin, that they do not evacuate air and blood effectively, and that blood clogs the valve. Provisional dressings using plastic (packaging) film appear to be generally unsuitable [73, 74]. Commercially available dressings, too, were reported to differ widely in efficacy [71–73]. Chest seals with laminar venting channels may be the most effective devices. [72]

### Limitations of the guideline

Patient values and preferences were sought but not received. The effect of this on the guideline is unclear, and there is a lack of research evidence on the effect of patient participation on treatment decisions or outcomes in the emergency setting.

## Supplementary Information

Below is the link to the electronic supplementary material.

Supplementary file1 (DOCX 43 KB)

## Data Availability

A full version of the guideline and its methods/evidence report are available online at https://register.awmf.org/de/leitlinien/detail/187-023

## Abbreviations

*AAL*: Anterior axillary line
*ABCDE*: Airway, breathing, circulation, disability, exposure
*AWMF*: German Association of the Scientific Medical Societies
*BMI*: Body mass index
*CI*: Confidence interval
*CT*: Computed tomography
*GPP*: Good Practice Point
*ICS*: Intercostal space
*LoE*: Level of evidence
*MAL*: Mid-axillary line
*MCL*: Mid-clavicular line
*MRI*: Magnetic resonance imaging
*PICO*: Population, intervention, comparison, outcome
*PRISMA*: Preferred Reporting Items for Systematic Reviews and Meta-Analyses
*RCT*: Randomised controlled trial
*TRISS*: Trauma and Injury Severity Score
