# Supplementary material for: Prehospital management of chest injuries in severely injured patients—a systematic review and clinical practice guideline update
Source: Eur J Trauma Emerg Surg. 2024 Feb 3;50(4):1367–80. doi: 10.1007/s00068-024-02457-3 (PMC11458653; doi:10.1007/s00068-024-02457-3)
Supplement: Supplementary file 1 — Supplementary file1 (DOCX 43 KB) [file 68_2024_2457_MOESM1_ESM.docx]

### Online Supplemental Material

# Prehospital management of chest injuries in severely injured patients – A systematic review and clinical practice guideline update

Waydhas C, et al

# S1 PICO Questions

|  | Population | Intervention(s) | Control(s) | Outcome(s) |
| --- | --- | --- | --- | --- |
| 1 | Target population | Clinical examination of the thorax and respiratory function, including determination of respiratory rate and auscultation of the lungs, follow-up. | None/not all of the above examinations | Patient-relevant outcomes |
| 2 | Target population | Inspection, palpation, percussion of the thorax, pulse oximetry and, in ventilated patients, monitoring of ventilation pressure and capnography. | No auscultation with regard to pneumothorax | Patient-relevant outcomes |
| 3 | Target population | Auscultation for normapnoea and thoracic analgesia to exclude a major pneumothorax (use of ultrasound diagnostics if necessary). | No monitoring of progressive pneumothoraces | Patient-relevant outcomes |
| 4 | Target population, with unilateral absence of breath sound on auscultation of the lung and the additional presence of typical symptoms, especially of severe respiratory or circulatory dysfunction. | Monitor for possible occult progressive pneumothoraces | No suspected diagnosis | Patient-relevant outcomes |
| 5 | Target population, with tension pneumothorax | Suspected diagnosis of tension pneumothorax (with therapeutic consequences) | Late (no) discharge | Patient-relevant outcomes |
| 6 | Target population, with tension pneumothorax under positive pressure ventilation | Early relief by decompression | No discharge, late discharge | Patient-relevant outcomes |
| 7 | Target population, with tension pneumothorax without ventilation | Relief by decompression | No close clinical monitoring | Patient-relevant outcomes |
| 8 | Target population, with tension pneumothorax | Close clinical monitoring | No relief by needle decompression or | Patient-relevant outcomes |
| 9 | Target population, with pneumothorax | Needle decompression followed by surgical opening of the pleural space with or without chest drainage, or | Needle decompression alone | Patient-relevant outcomes |
| 10^§^ | Target population, with haematothorax, haematopneumothorax or pneumothorax | Needle decompression, followed by surgical opening of the pleural space with or without chest drainage if not effective enough. | No chest drainage | Patient-relevant outcomes |
| 11 | Target population, with pneumothorax | Chest drainage | Large lumen drainage (>24 Ch) | Patient-relevant outcomes |
| 12 | Target population, with pneumothorax | Small lumen drainage (≤24Ch) | Other opening of the pleural space | Patient-relevant outcomes |

# S2 Literature Search

| Suchstrategie 2021, MEDLINE (via Ovid) Datum: 19.05.2021 3.241 Treffer |
| --- |
| 1. exp Pneumothorax/ or exp hemopneumothorax/ or exp thoracic injuries/ or exp Thorax/ or exp Cardiac Tamponade/ or exp Lung Injury/  2. (pneumothora* or h?emopneumothora* or h?emothorax or thora* injur* or chest injur* or thora* trauma* or chest trauma* or myocardial injur* or myocardial trauma* or heart tamponade or cardiac tamponade or lung injur* or lung trauma* or tracheobronchial injur* or tracheobronchial trauma*).ab,ti,kf.  3. 1 or 2  4. exp Multiple Trauma/  5. (polytrauma* or trauma patient?).ti,ab,kf. or (severe adj2 shock).ti,ab,kf.  6. ((multiple or major or severe* or serious*) adj3 (trauma* or injur*)).ti,ab,kf.  7. ((blunt or penetrating) adj5 (trauma* or injur*)).ti,ab,kf.  8. (*Critical Care/ or *Emergencies/ or (life threatening or critical care or emergen*).ti,ab,kf.) and (trauma* or injur*).ti,ab,kf.  9. 4 or 5 or 6 or 7 or 8  10. 3 and 9  11. exp animals/ not humans.sh.  12. 10 not 11  13. (comment or editorial or letter).pt. or case report*.mp.  14. 12 not 13  15. limit 14 to dt=20140601-20210531 |
| Suchstrategie 2021, Embase (via Elsevier) Datum: 19.05.2021 1.407 Treffer |
| #1 'pneumothorax'/exp OR 'hematopneumothorax'/exp OR 'thorax injury'/exp OR 'thorax'/exp OR 'heart tamponade'/exp OR 'lung injury'/exp  #2 (pneumothora* OR h?emopneumothora* OR h?emothorax OR "thora* injur*" OR "chest injur*" OR "thora* trauma*" OR "chest trauma*" OR “myocardial injur*” OR “myocardial trauma*” OR “heart tamponade” OR “cardiac tamponade” OR “lung injur*” OR “lung trauma*” OR “tracheobronchial injur*” OR “tracheobronchial trauma*”):ti,ab,kw  #3 #1 OR #2  #4 'multiple trauma'/exp  #5 (polytrauma* OR "trauma patient?"):ti,ab,kw OR (severe NEXT/2 shock):ti,ab,kw  #6 ((multiple OR major OR severe* OR serious*) NEXT/3 (trauma* OR injur*)):ti,ab,kw  #7 ((blunt OR penetrating) NEXT/5 (trauma* OR injur*)):ti,ab,kw  #8 ('intensive care'/mj OR 'emergency'/mj OR ("life threatening" OR "critical care" OR emergen*):ti,ab,kw) AND (trauma* OR injur*):ti,ab,kw  #9 #4 OR #5 OR #6 OR #7 OR #8  #10 #3 AND #9  #11 'animals'/exp NOT 'humans'/de  #12 #10 NOT #11  #13 (comment OR editorial OR letter):it OR "case report*":ti,ab,kw  #14 #12 NOT #13  #15 [1-6-2014]/sd NOT [1-6-2021]/sd  #16 #14 AND #15  #17 [embase]/lim  #18 #16 AND #17  #19 embase NOT (embase AND medline)  #20 #18 AND #19  #21 #20 AND ('article'/it OR 'article in press'/it OR 'erratum'/it OR 'review'/it) |

# S3 Excluded Studies

| **Reference** | **Reason for exclusion** |
| --- | --- |
| Abbasi S, Shaker H, Zareiee F, Farsi D, Hafezimoghadam P, Rezai M, et al. Screening performance of Ultrasonographic B-lines in Detection of Lung Contusion following Blunt Trauma; a Diagnostic Accuracy Study. Emergency (Tehran, Iran). 2018;6(1):Keine Mehrfachpublikation ohne Zusatzinformationen5. | Population |
| Abd El-Hafez Fouly M, Zahra A, Ghalwash M. Thoracoscopy versus thoracotomy in hemodynamically stable patients with closed thoracic trauma. Journal of the Egyptian Society of Cardio-Thoracic Surgery. 2018;26(1):64-7. | Population |
| Abdulrahman Y, Musthafa S, Hakim SY, Nabir S, Qanbar A, Mahmood I, et al. Utility of extended FAST in blunt chest trauma: is it the time to be used in the ATLS algorithm? World Journal of Surgery. 2015;39(1):172-8. | Population |
| Abedi Khorasgani M, Shahrami A, Shojaee M, Alimohammadi H, Amini A, Hatamabadi HR. The Accuracy of Plain Radiography in Detection of Traumatic Intrathoracic Injuries. Emergency (Tehran, Iran). 2016;4(4):184-7. | Population |
| Augustin P, Guivarch E, Tran-Dinh A, Pellenc Q, Tanaka S, Montravers P. Usefulness of CT-scan in the management of chest stab trauma: a prospective observational study. European Journal of Trauma & Emergency Surgery. 2020;46(6):1385-91. | Population |
| Azizi N, ter Avest E, Hoek AE, Admiraal-van de Pas Y, Buizert PJ, Peijs DR, et al. Optimal anatomical location for needle chest decompression for tension pneumothorax: A multicenter prospective cohort study. Injury. 2021;52(2):213-8. | Population |
| Bar A, Lin G, Lazar LO, Blanka-Deak J, Khalayleh H, Pines G. Immediate Pneumothorax Diagnosis by Surgical Residents Using Portable Ultrasound. Innovations: Technology & Techniques in Cardiothoracic & Vascular Surgery. 2021;16(2):152-6. | Population |
| Bauman ZM, Kulvatunyou N, Joseph B, Jain A, Friese RS, Gries L, et al. A Prospective Study of 7-Year Experience Using Percutaneous 14-French Pigtail Catheters for Traumatic Hemothorax/Hemopneumothorax at a Level-1 Trauma Center: Size Still Does Not Matter. World Journal of Surgery. 2018;42(1):107-13. | Study type |
| Bhoil R, Kumar R, Kaur J, Attri PK, Thakur R. Diagnosis of Traumatic Pneumothorax: A Comparison between Lung Ultrasound and Supine Chest Radiographs. Indian Journal of Critical Care Medicine. 2021;25(2):176-80. | Population |
| Billeter, A. T., et al. (2013). "Video-assisted thoracoscopy as an important tool for trauma surgeons: a systematic review." Langenbecks Archives of Surgery 398(4): 515-523. | Study type |
| Burrell AJ, Kaye DM, Fitzgerald MC, Cooper DJ, Hare JL, Costello BT, et al. Cardiac magnetic resonance imaging in suspected blunt cardiac injury: A prospective, pilot, cohort study. Injury. 2017;48(5):1013-9. | Intervention |
| Carter R, Wee IJY, Petrie K, Syn N, Choong AM. Chimney parallel grafts and thoracic endovascular aortic repair for blunt traumatic thoracic aortic injuries: A systematic review. Vascular. 2019;27(2):204-12. | Study type |
| Chan KK, Joo DA, McRae AD, Takwoingi Y, Premji ZA, Lang E, et al. Chest ultrasonography versus supine chest radiography for diagnosis of pneumothorax in trauma patients in the emergency department. Cochrane Database of Systematic Reviews. 2020;7:CD013031. | Multiple publication |
| Dabees NL, Salama AA, Elhamid SA, Sabry MM. Multi-detector computed tomography imaging of blunt chest trauma. Egyptian Journal of Radiology and Nuclear Medicine. 2014;45(4):1105-13. | Population |
| Ebrahimi A, Yousefifard M, Mohammad Kazemi H, Rasouli HR, Asady H, Moghadas Jafari A, et al. Diagnostic Accuracy of Chest Ultrasonography versus Chest Radiography for Identification of Pneumothorax: A Systematic Review and Meta-Analysis. Tanaffus. 2014;13(4):29-40. | Population |
| Emet M, Akoz A, Aslan S, Saritas A, Cakir Z, Acemoglu H. Assessment of cardiac injury in patients with blunt chest trauma. European Journal of Trauma & Emergency Surgery. 2010;36(5):441-7. | Population |
| Gilbert RW, Fontebasso AM, Park L, Tran A, Lampron J. The management of occult hemothorax in adults with thoracic trauma: A systematic review and meta-analysis. The Journal of Trauma and Acute Care Surgery. 2020;89(6):1225-32. | Study type |
| Gonzalez-Hadad A, Garcia AF, Serna JJ, Herrera MA, Morales M, Manzano-Nunez R. The Role of Ultrasound for Detecting Occult Penetrating Cardiac Wounds in Hemodynamically Stable Patients. World Journal of Surgery. 2020;44(5):1673-80. | Population |
| Helmy S, Beshay B, Abdel Hady M, Mansour A. Role of chest ultrasonography in the diagnosis of lung contusion. Egyptian Journal of Chest Diseases and Tuberculosis. 2015;64(2):469-75. | Population |
| Heydari F, Esmailian M, Dehghanniri M. Diagnostic Accuracy of Ultrasonography in the Initial Evaluation of Patients with Penetrating Chest Trauma. Emergency (Tehran, Iran). 2014;2(2):81-4. | Population |
| Hosseini M, Ghelichkhani P, Baikpour M, Tafakhori A, Asady H, Haji Ghanbari MJ, et al. Diagnostic Accuracy of Ultrasonography and Radiography in Detection of Pulmonary Contusion; a Systematic Review and Meta-Analysis. Emergency (Tehran, Iran). 2015;3(4):127-36. | Study type |
| Jahanshir A, Moghari SM, Ahmadi A, Moghadam PZ, Bahreini M. Value of point-of-care ultrasonography compared with computed tomography scan in detecting potential life-threatening conditions in blunt chest trauma patients. The Ultrasound Journal. 2020;12(1):36. | Population |
| Jin J, Song B, Lei YC, Leng XF. Video-assisted thoracoscopic surgery for penetrating thoracic trauma. Chinese Journal of Traumatology. 2015;18(1):39-40. | Population |
| Karacabey S, Sanri E, Metin B, Erkoc F, Yildirim S, Intepe YS, et al. Use of ultrasonography for differentiation between bullae and pneumothorax. Emergency Radiology. 2019;26(1):15-9. | Population |
| Kaya S, Cevik AA, Acar N, Doner E, Sivrikoz C, Ozkan R. A study on the evaluation of pneumothorax by imaging methods in patients presenting to the emergency department for blunt thoracic trauma. Ulusal Travma ve Acil Cerrahi Dergisi = Turkish Journal of Trauma & Emergency Surgery: TJTES. 2015;21(5):366-72. | Population |
| Khoynezhad A, Donayre CE, Azizzadeh A, White R, investigators R. One-year results of thoracic endovascular aortic repair for blunt thoracic aortic injury (RESCUE trial). Journal of Thoracic & Cardiovascular Surgery. 2015;149(1):155-61.Publikationssprache. | Study type |
| Kondo Y, Ohbe H, Yasunaga H, Tanaka H. Initial focused assessment with sonography in trauma versus initial CT for patients with haemodynamically stable torso trauma. Emergency Medicine Journal. 2020;37(1):19-24. | Population |
| Kumar S, Agarwal N, Rattan A, Rathi V. Does intrapleural length and position of the intercostal drain affect the frequency of residual hemothorax? A prospective study from north India. Journal of Emergencies Trauma & Shock. 2014;7(4):274-9. | Population |
| Langdorf MI, Medak AJ, Hendey GW, Nishijima DK, Mower WR, Raja AS, et al. Prevalence and Clinical Import of Thoracic Injury Identified by Chest Computed Tomography but Not Chest Radiography in Blunt Trauma: Multicenter Prospective Cohort Study. Annals of Emergency Medicine. 2015;66(6):589-600. | Population |
| Li G, Wu XW, Lu WH, Cheng J, Wu XY, Ai R, et al. High-sensitivity cardiac troponin T: A biomarker for the early risk stratification of type-A acute aortic dissection? Archives of cardiovascular diseases. 2016;109(3):163-70. | Population |
| Macri F, Greffier J, Khasanova E, Claret PG, Bastide S, Larbi A, et al. Minor Blunt Thoracic Trauma in the Emergency Department: Sensitivity and Specificity of Chest Ultralow-Dose Computed Tomography Compared With Conventional Radiography. Annals of Emergency Medicine. 2019;73(6):665-70. | Population |
| Manzano-Nunez R, Gomez A, Espitia D, Sierra-Ruiz M, Gonzalez J, Rodriguez-Narvaez JG, et al. A meta-analysis of the diagnostic accuracy of chest ultrasound for the diagnosis of occult penetrating cardiac injuries in hemodynamically stable patients with penetrating thoracic trauma. The Journal of Trauma and Acute Care Surgery. 2021;90(2):388-95. | Multiple publication |
| Mishra PR, Bhoi S, Sinha TP. Integration of Point-of-care Ultrasound during Rapid Sequence Intubation in Trauma Resuscitation. Journal of Emergencies Trauma & Shock. 2018;11(2):92-7. | Intervention |
| Morales CH, Mejia C, Roldan LA, Saldarriaga MF, Duque AF. Negative pleural suction in thoracic trauma patients: A randomized controlled trial. The Journal of Trauma and Acute Care Surgery. 2014;77(2):251-5. | Population |
| Moussavi N, Davoodabadi AH, Atoof F, Razi SE, Behnampour M, Talari HR. Routine chest computed tomography and patient outcome in blunt trauma. Archives of Trauma Research. 2015;4(2). | Population |
| Reichardt GS, Nogueira GM, Rafael LK, Soltoski PR, Pimentel SK. CT scanning in blunt chest trauma: validation of decision instruments. Revista do Colegio Brasileiro de Cirurgioes. 2020;47:Intervention0202648. | Population |
| Rodriguez RM, Baumann BM, Raja AS, Langdorf MI, Anglin D, Bradley RN, et al. Diagnostic yields, charges, and radiation dose of chest imaging in blunt trauma evaluations. Academic Emergency Medicine. 2014;21(6):644-50. | Population |
| Rodriguez RM, Canseco K, Baumann BM, Mower WR, Langdorf MI, Medak AJ, et al. Pneumothorax and Hemothorax in the Era of Frequent Chest Computed Tomography for the Evaluation of Adult Patients With Blunt Trauma. Annals of Emergency Medicine. 2019;73(1):58-65. | Study type |
| Samuel AE, Chakrapani A, Moideen F. Accuracy of Extended Focused Assessment with Sonography in Trauma (e-FAST) Performed by Emergency Medicine Residents in a Level One Tertiary Center of India. Advanced Journal of Emergency Medicine. 2018;2(2):Population5. | Population |
| Schellenberg M, Inaba K, Bardes JM, Orozco N, Chen J, Park C, et al. The combined utility of extended focused assessment with sonography for trauma and chest x-ray in blunt thoracic trauma. The Journal of Trauma and Acute Care Surgery. 2018;85(1):113-7. | Population |
| Staub LJ, Biscaro RRM, Kaszubowski E, Maurici R. Chest ultrasonography for the emergency diagnosis of traumatic pneumothorax and haemothorax: A systematic review and meta-analysis. Injury. 2018;49(3):457-66. | Multiple publication |
| Stengel D, Leisterer J, Ferrada P, Ekkernkamp A, Mutze S, Hoenning A. Point-of-care ultrasonography for diagnosing thoracoabdominal injuries in patients with blunt trauma. Cochrane Database of Systematic Reviews. 2018;12:CD012669. | Population |
| Tunuka CE, Wangoda R, Bugeza S, Galuk, e M. Emergency sonography aids diagnostic accuracy of torso injuries: A study in a resource limited setting. Emergency Medicine International. 2014;2014. | Population |
| Vafaei A, Hatamabadi HR, Heidary K, Alimohammadi H, Tarbiyat M. Diagnostic Accuracy of Ultrasonography and Radiography in Initial Evaluation of Chest Trauma Patients. Emergency (Tehran, Iran). 2016;4(1):29-33. | Population |
| van der Zee CP, Vainas T, van Brussel FA, Tielliu IF, Zeebregts CJ, van der Laan MJ. Endovascular treatment of traumatic thoracic aortic lesions: a systematic review and meta-analysis. Journal of Cardiovascular Surgery. 2019;60(1):100-10. | Study type |
| van Rein EAJ, Lokerman RD, van der Sluijs R, Hjortnaes J, Lichtveld RA, Leenen LPH, et al. Identification of thoracic injuries by emergency medical services providers among trauma patients. Injury. 2019;50(5):1036-41. | Population |
| Wu N, Wu L, Qiu C, Yu Z, Xiang Y, Wang M, et al. A comparison of video-assisted thoracoscopic surgery with open thoracotomy for the management of chest trauma: a systematic review and meta-analysis. World Journal of Surgery. 2015;39(4):940-52. | Study type |
| Yates, J. G. and D. Baylous (2017). "Aeromedical Ultrasound: The Evaluation of Point-of-care Ultrasound During Helicopter Transport." Air Medical Journal 36(3): 110-115. | Population |
| Billeter, A. T.; Druen, D.; Franklin, G. A.; Smith, J. W.; Wrightson, W.; Richardson, J. D.; Video-assisted thoracoscopy as an important tool for trauma surgeons: a systematic review. Langenbecks Archives of Surgery - Volume 398, Issue 4, pp. 515-23 | Study type |
| Bouillon B, Marzi I (2018) The updated German "Polytrauma - Guideline": an extensive literature evaluation and treatment recommendation for the care of the critically injured patient. Eur J Trauma Emerg Surg 44:1 | Study type |
| Chang SH, Kang YN, Chiu HY et al. (2018) A Systematic Review and Meta-Analysis Comparing Pigtail Catheter and Chest Tube as the Initial Treatment for Pneumothorax. Chest 153:1201-1212 | Study type |
| Laan DV, Vu TD, Thiels CA et al. (2016) Chest wall thickness and decompression failure: A systematic review and meta-analysis comparing anatomic locations in needle thoracostomy. Injury 47:797-804 | Study type |
| Maezawa T, Yanai M, Huh JY et al. (2020) Effectiveness and safety of small-bore tube thoracostomy (</=20 Fr) for chest trauma patients: A retrospective observational study. Am J Emerg Med 38:2658-2660 | Study type |
| Tanizaki S, Maeda S, Sera M et al. (2017) Small tube thoracostomy (20-22 Fr) in emergent management of chest trauma. Injury 48:1884-1887 | Study type |
| Video-Assisted Thoracoscopic Surgery in theTreatment of Chest Trauma: Long-Term BenefitAlon Ben-Nun,MD, PhD,Michael Orlovsky,MD,and Lael Anson Best,MDDepartment of General Thoracic Surgery, Rambam Medical Center, Haifa, Israel | Study type |
| Video-assisted thoracoscopic surgery for acute thoracic trauma Michael Goodman,Jaime Lewis,Julian Guitron,Michael Reed,Timothy Pritts, and Sandra Starnes | Study type |
| Randomized clinical trial of pigtail catheterversuschest tubein injured patients with uncomplicated traumatic pneumothoraxN. Kulvatunyou, L. Erickson, A. Vijayasekaran, L. Gries, B. Joseph, R. F. Friese, T. O’Keeffe,A. L. Tang, J. L. Wynne and P. Rhee | Population |
| Clements TW, Sirois M, Parry N, Roberts DJ, Trottier V, Rizoli S, et al. OPTICC: A multicentre trial of Occult Pneumothoraces subjected to mechanical ventilation: The final report. American Journal of Surgery. 2021;20:20. | No matching PICO |
| Kozaci N, Avci M, Ararat E, Pinarbasili T, Ozkaya M, Etli I, et al. Comparison of ultrasonography and computed tomography in the determination of traumatic thoracic injuries. American Journal of Emergency Medicine. 2019;37(5):864-8. | No matching PICO |
| Kulvatunyou N, Bauman ZM, Edine SBZ, de Moya M, Krause C, Mukherjee K, et al. The Small 14-French (Fr) Percutaneous Catheter vs. Large (28-32Fr) Open Chest Tube for Traumatic Hemothorax (P-CAT): A Multi-center Randomized Clinical Trial. The Journal of Trauma and Acute Care Surgery. 2021;16:16. | No matching PICO |
| Lang P, Kulla M, Kerwagen F, Lefering R, Friemert B, Palm HG, et al. The role of whole-body computed tomography in the diagnosis of thoracic injuries in severely injured patients - a retrospective multi-centre study based on the trauma registry of the German trauma society (TraumaRegister DGU<sup> R</sup>). Scandinavian Journal of Trauma, Resuscitation & Emergency Medicine. 2017;25(1):82. | No matching PICO |
| Leblanc D, Bouvet C, Degiovanni F, Nedelcu C, Bouhours G, Rineau E, et al. Early lung ultrasonography predicts the occurrence of acute respiratory distress syndrome in blunt trauma patients. Intensive Care Medicine. 2014;40(10):1468-74. | No matching PICO |
| Mahmood I, Tawfeek Z, El-Menyar A, Zarour A, Afifi I, Kumar S, et al. Outcome of concurrent occult hemothorax and pneumothorax in trauma patients who required assisted ventilation. Emergency Medicine International Print. 2015;2015:859130. | No matching PICO |
| Matsumoto S, Sekine K, Funabiki T, Orita T, Shimizu M, Hayashida K, et al. Diagnostic accuracy of oblique chest radiograph for occult pneumothorax: comparison with ultrasonography. World Journal Of Emergency Surgery. 2016;11:5. | No matching PICO |
| Ojaghi Haghighi SH, Adimi I, Shams Vahdati S, Sarkhoshi Khiavi R. Ultrasonographic diagnosis of suspected hemopneumothorax in trauma patients. Trauma Monthly. 2014;19(4):e17498. | No matching PICO |
| Rodriguez RM, Friedman B, Langdorf MI, Baumann BM, Nishijima DK, Hendey GW, et al. Pulmonary contusion in the pan-scan era. Injury. 2016;47(5):1031-4. | No matching PICO |
| Zachary M. Bauman1•Narong Kulvatunyou2•Bellal Joseph2•Lynn Gries2•Terence O’Keeffe2•Andrew L. Tang2•Peter Rhee3 Randomized Clinical Trial of 14-French (14F) Pigtail Cathetersversus28–32F Chest Tubes in the Management of Patientswith Traumatic Hemothorax and Hemopneumothorax | No matching PICO |
| Aiolfi A, Inaba K, Martin M, Matsushima K, Bonitta G, Bona D, et al. Lung Resection for Trauma: A Propensity Score Adjusted Analysis Comparing Wedge Resection, Lobectomy, and Pneumonectomy. American Surgeon. 2020;86(3):261-5. | No matching PICO |
| Finnegan P, Fitzgerald M, Smit D, Martin K, Mathew J, Varma D, et al. Video-tube thoracostomy in trauma resuscitation: A pilot study. Injury. 2019;50(1):90-5. | No matching PICO |
| Huang, W. Y., et al. (2016). "Efficiency Analysis of Direct Video-Assisted Thoracoscopic Surgery in Elderly Patients with Blunt Traumatic Hemothorax without an Initial Thoracostomy." BioMed Research International 2016: 3741426. | No matching PICO |

# S4 Evidence Table

##### Suspected diagnosis of pneumothorax and/or hematothorax

| Study: Reference, aim, design, setting | Participants: selection criteria, characteristics | N Participants; Intervention (IG) vs. Control group (CG) | Main outcomes | Assessment: LoE, risk of bias; Conclusions |
| --- | --- | --- | --- | --- |
| Press (2014)  "Prospective Evaluation of Prehospital Trauma Ultrasound During Aeromedical Transport". *The Journal of Emergency Medicine* 2014*,* Vol. 47, No. 6, pp. 638–645.  Study design  Diagnostic cross-sectional study  Aim of the study  “The goal was to assess prehospital provider accuracy in performing the abdominal, cardiac, and lung components of EFAST.”  Setting  USA, 7-month-study | Inclusion criteria   - adult trauma patients (18 years or older) transferred directly from scene if time allowed after standard stabilization   Exclusion criteria  NR  Characteristics  Age [y], mean ± SD  41 ± 17  Male, n (%)  216 (74)  ISS mean ± SD  16 ± 11  Trauma type, n (%)  Blunt 252 (88.4)  Penetrating 33 (11.6)  Weight (kg), mean ± SD  82 ± 18  Scene systolic blood pressure (mm Hg), mean ± SD  130 ± 27  Scene heart rate (bpm), mean ± SD  94 ± 22  Base deficit, mean ± SD  3.1 ± 4.5  Transport time to ED (min), mean ± SD  20.9 ± 8.7  ED GCS  12 + 3/–4 | Participants  Adult trauma patients from scene N=833  Patients with at least one HEMS ultrasound n=293  Number of lung HEMS ultrasound n=511  Tests evaluated  Index text: In flight ultrasound. HEMS providers were trained to perform EFAST during a 2-month period. HEMS providers performed EFAST using the following views: hepatorenal, splenorenal, suprapubic, cardiac (subcostal or parasternal long-axis), right lung, and left lung. All views were standard and in accordance with imaging described by the American College of Emergency Physicians and American Institute of Ultrasound in Medicine (19). Abdominal and cardiac examinations were performed to evaluate for intraperitoneal and pericardial fluid, respectively. Lung ultrasound was performed to evaluate for lung slide to exclude or diagnose pneumothorax. Abdominal views were saved as still images, and cardiac and lung views as 4-s video clips.  Reference standard: ED diagnostics and management including CT, chest radiography and clinical examination. | Diagnostic test performance  Lung Pneumothorax  true positive, n=8  false positive, n=2  true negative, n=444  false negative, n=35  sensitivity, % (95% CI), n/N  18.7 (8.9–33.9), 8/43  specificity, % (95% CI) , n/N  99.5 (98.2–99.9), 444/446  PPV, % (95% CI) , n/N  80 (44.2–96.5), 8/10  NPV, % (95% CI) , n/N  92.7 (89.9–94.8), 444/479  Lung Pneumothorax required intervention  true positive, n=9  false positive, N=1  true negative, n=469  false negative, n=0  sensitivity, % (95% CI) , n/N  50 (22.3–58.7), 9/9  specificity, % (95% CI) , n/N  99.8 (98.6–100), 469/470  PPV, % (95% CI) , n/N  90 (54.1–99.5), 9/10  NPV, % (95% CI) , n/N  98.1 (96.3–99.1), 469/478 | Level of evidence  2b  Risk of bias  Patient selection: +  Index test: +  Reference standard: +  Flow and timing: +  Authors’ conclusion  “Positive interpretations significantly raised the probability of injury, more reliably so for lung ultrasound. Negative interpretations were predictive, but low prevalence limited the value of these results. Sensitivity was not sufficient for ruling out injury. We believe further study is needed to elucidate accuracy as providers gain experience, and to explore clinical outcomes that may be affected by prehospital trauma ultrasound.”  Reviewers’ conclusion  HEMS providers were new to inflight ultrasound and received a training. The guidance from this training may have a high influence on current behaviour. Also all staff knew about the study which may have introduced a Hawthorne effect. |
| Quick (2016)  "In-flight ultrasound identification of pneumothorax". *Emerg Radiol* (2016) 23:3–7.  Study design  Diagnostic cross-sectional study  Aim of the study  “Our study sought to demonstrate the accurate and timely detection of correctable thoracic pathology, specifically pneumothorax and improperly positioned endotracheal tubes by non-physician, prehospital flight crews trained in the use of thoracic ultrasound.”  Setting  USA, 15-month | Inclusion criteria   - all adult trauma patients, - all intubated adult medical patients transported by one of University of Missouri’s Staff for Life Helicopters   Exclusion criteria  NR  Characteristics  Age [y], mean (range)  44.4 (16-94)  Male, n (%)  133 (69)  ISS mean (range)  17.68 (1-75)  Chest AIS mean (range)  2.93 (0-6)  BMI mean (range)  28.2 (15–50)  149 patients (136 trauma/13 medical) met inclusion criteria. | Participants  N=149 In-flight ultrasound  N=116 CT scan  Tests evaluated  Index text: In-flight ultrasound. Twenty-six flight crew members were trained to perform and interpret thoracic ultrasound prior to the initiation of the study. Flight crews recorded their interpretations of radiographic findings using an evaluation form.  Reference standard: CT scan. Routine clinical care was provided in accordance with ATLS methods to include the completion of an E-FAST by the trauma team. Further imaging was obtained as needed during patient evaluation. Computed tomography (CT) was considered the criterion standard and utilized to confirm either the presence or absence of pneumothorax and proper endotracheal tube placement. Patients that did not undergo CT evaluation had either clearly visible pneumothorax on chest X-ray or definitive clinical signs of a pneumothorax. | Diagnostic test performance  Lung Pneumothorax  true positive, n=16  false positive, n=1  true negative, n=129  false negative, n=3  sensitivity, % (95% CI)  68 (0.46–0.85)  specificity, % (95% CI)  96% (CI 0.90–0.98)  PPV, % (95% CI)  NR  NPV, % (95% CI)  NR  **Diagnostic accuracy, % (95% CI)**  91 (0.85–0.95)  Right lung ultrasound was more sensitive than left for both pneumothorax (18.7% vs. 8.7%) and required intervention (66.6% vs.33.3%). | Level of evidence  2b  Risk of bias  Patient selection: +  Index test: +  Reference standard: +  Flow and timing: ?  Authors’ conclusion  “Ultrasonography should be utilized to augment the diagnostic capabilities of all prehospital aeromedical providers. Routine use of in-flight ultrasound is one step closer to getting the right care to the right patient at the earliest possible instance and could lead to better outcomes. A multicenter trial is warranted to further confirm this benefit.”  Reviewers’ conclusion  Providers were new to inflight ultrasound and received a training. The guidance from this training may have a high influence on current behaviour. It is unclear how many patients received chest radiography as reference standard. |
| +: low risk; –: high risk; ?: unclear risk; adj.: adjusted; AIS: abbreviated injury score; ATLS: Advanced Trauma Life Support; BMI: body mass index; CG: control group; CI: Confidence Interval; CT: computed tomography; d: days; ED: emergency department; (e)FAST: extended version of the Focused Assessment with Sonography for Trauma; GCS: Glasgow coma scale; HR: Hazard Ratio; HEMS: helicopter emergency medical services; IG: intervention group; IQR: Interquartile Range; ISS: injurie severity score; ITT: Intention to Treat; LoE: level of evidence; m: months; NPV: negative predictive value; NR: not reported; OR: Odds Ratio; PPV: positive predictive value; RR: Relative Risk; SD: Standard Deviation; SEM: Standard Error of Mean; y: years | | | | |

# S5 Deleted Recommendations

| 1.5 | Thorax |  |  |  |
| --- | --- | --- | --- | --- |
| 1.30 0 | Bei hypotonen Patienten mit schwerem Schädel-Hirn-Trauma kann eine hypertone Lösung verwendet werden. | - | - | Empfehlung gestrichen (EK) |
| 1.37  A | Der Spannungspneumothorax ist die häufigste reversible Ursache des traumatischen Herzkreis-laufstillstandes und soll in der Präklinik entlastet werden.  *Neu 2016* | - | - | Empfehlung gestrichen |
